# Cerebrovascular Consequences of Obstructive Sleep Apnea

**DOI:** 10.1161/JAHA.111.000091

**Published:** 2012-08-24

**Authors:** David J. Durgan, Robert M. Bryan

**Affiliations:** Department of Anesthesiology, Baylor College of Medicine, Houston, TX (D.J.D., R.M.B.); Department of Molecular Physiology and Biophysics, Baylor College of Medicine, Houston, TX (R.M.B.); Department of Medicine (Cardiovascular Sciences), Baylor College of Medicine, Houston, TX (R.M.B.)

**Keywords:** cerebrovascular circulation, cerebrovascular disease, dementia, sleep apnea, obstructive, sleep apnea syndromes, stroke, ischemic attack, transient

## Introduction

**Figure 1.**
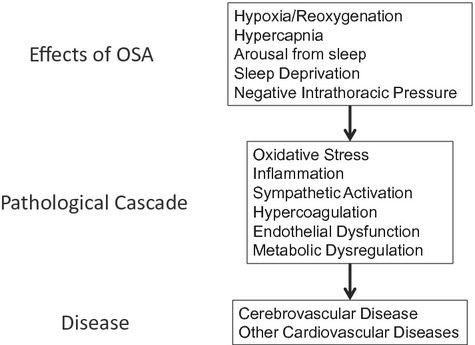
The effects of OSA lead to a pathological cascade that is responsible for cerebrovascular and other cardiovascular diseases. Adapted from Somers et al.^1^

Obstructive sleep apnea (OSA) is defined by interrupted breathing during sleep due to airway obstruction with an ongoing respiratory effort. In adults, OSA most commonly is caused by decreased muscle tone (required for patency) in the soft tissues of the upper airway.^1^ Symptoms of OSA include snoring, daytime sleepiness, morning headache, sexual dysfunction, and mood and behavioral disorders.^1^ Although sometimes overlooked by physicians, OSA is an independent predictor for cardiovascular disorders.^1–7^ In an effort to educate physicians about the seriousness of sleep apnea, the American Heart Association and American College of Cardiology issued a joint statement in 2008 with the purpose of communicating the idea that sleep apnea is the underlying cause of cardiovascular disease in some patients, whereas it exacerbates this pathological condition in others.^1^ Cardiovascular diseases associated with OSA include hypertension, heart failure, stroke, cardiac arrhythmias, myocardial ischemia and infarction, and pulmonary arterial hypertension.^1–3,5,7^ In addition, OSA is associated with metabolic dysregulation (insulin resistance and lipid disorders), which in turn is a risk factor for cardiovascular diseases.^1,3,7^ Figure 1 shows characteristics of OSA and the pathological cascade that can lead to cardiovascular diseases. In this review, our focus will be the effects of OSA on the cerebral circulation and on cerebrovascular diseases. Central sleep apnea, another form of sleep-disordered breathing that is defined by cessation of breathing due to a loss of ventilatory drive, will not be covered except when pertinent to the discussion of OSA.

### Definitions and Classification

The result of excessive airway collapse is apnea, a cessation of airflow for >10 seconds, or hypopnea, a reduction of airflow to <50% of normal, or both.^7^ Although the definitions of apnea and hypopnea can vary, they generally do not deviate significantly from those given in the previous sentence. OSA is graded on a scale termed the apnea-hypopnea index (AHI), which consists of the mean number of apneas and hypopneas per hour of sleep. An AHI of 5 to 15 is considered mild, 16 to 30 is considered moderate, and >30 is considered severe.^1^ Of note, AHI in severe OSA can exceed 100. A fall in O_2_ saturation of 10% to 15% is common during an OSA event; however, oxygen saturation can fall by ≥50% in extreme cases.^1^ Importantly, a decrease in the O_2_ saturation of hemoglobin by only 4% during OSA is associated with an increased incidence of cardiovascular disease independent of confounding covariates.^8^

### Epidemiology of OSA

Estimates of the incidence of OSA vary from <5% to up to 25% of the adult population in the Western world.^1–3,9–12^ OSA almost certainly will become an even greater health problem in the future because 2 of the prominent risk factors for OSA, obesity and older age, are on the rise.^1,3,11,13–15^ Of further significance to the public health dilemma are the facts that (1) ≍85% of people with clinically significant OSA go undiagnosed, and (2) OSA can continue for years without the individual being aware of it.^1^

### Treatment for OSA

The major treatment for OSA involves continuous positive airway pressure (CPAP), a therapy that holds the airway open by using pressures delivered by a mask. Variations that use positive pressure currently are being used, but all maintain positive pressure during inhalation. Although the use of CPAP seems to be beneficial in some studies, more randomized, prospective studies are needed to fully clarify the effectiveness of CPAP.^16^ Unfortunately, 30% of all patients with OSA do not tolerate CPAP or do not comply because of the discomfort of the CPAP mask, which is strapped over the nose or over both the nose and mouth by a headband. In asymptomatic or minimally symptomatic patients, lack of compliance is even greater.^16^ This lack of compliance is compounded by the estimate that 93% of women and 82% of men with moderate to severe sleep apnea syndrome have not been clinically diagnosed.^17^ Other therapies, such as mandibular devices, weight reduction, and surgery, have limited effectiveness in milder forms of OSA but generally are not effective in more severe OSA.^16^ Pharmacological therapies have been disappointing, even though several dozen drugs have been evaluated for the treatment of OSA.^16^

## OSA and Cerebrovascular Disease

### OSA and Stroke

The incidence of OSA in patients who have had a stroke or transient ischemic attack is greater than that of the general population.^18^ Patients presenting with stroke or transient ischemic attack were 3 to 4 times more likely to have OSA than were matched control subjects.^19–20^ Regardless of sex, between 60% and 80% of patients with stroke and transient ischemic attack had an AHI >10.^20–22^ Furthermore, the incidence of obstructive apnea was ≍7-fold greater than the incidence of apneas that were predominantly central in nature.^21–22^ Palomaki^23^ compared 177 consecutive male patients with brain infarctions to age-matched controls. The odds ratio for snoring and brain infarction was 2.13 (95% confidence interval [CI], 1.29 to 3.52). (*Odds ratio* is defined as the odds of an event occurring in one group divided by the odds of the same event occurring in another group.) If sleep apnea (reported by subject or partner), daytime sleepiness, and obesity were also present, the odds ratio increased to 8.0 (95% CI, 1.07 to 356.1). Palomaki concluded that snoring could be a risk factor for ischemic stroke, likely because of the higher prevalence of OSA among snorers. Although the aforementioned studies are revealing and provocative, they did not determine definitively whether OSA was present before stroke or was the result of stroke.

Cross-sectional and longitudinal studies involving stroke and OSA confirm and amplify the findings of the previously discussed studies.^18^ Redline et al^24^ monitored 5422 participants from the Sleep Heart Health Study over a median of 8.7 years. Participants were excluded if they had a history of stroke at the baseline examination (1995–1998) or if they had been treated for OSA. A significant positive association between AHI and ischemic stroke in men (*P*=0.016) was observed. For men in the highest quartile (AHI >19), the hazard ratio was 2.86 (95% CI, 1.1 to 7.4) after age, body mass index, smoking status, systolic blood pressure, use of antihypertensive medications, diabetes mellitus, and race were incorporated into the model. (The *hazard ratio* is similar to the odds ratio except it involves not only the occurrence but also the rates at which an event occurs.) The stroke risk was estimated to increase by 6% for each 1-unit increase in AHI between 5 and 25. In contrast to men, the incidence for stroke was not significantly associated with AHI quartiles in women. Still, an increased risk was observed in women when AHI was >25. Redline et al pointed out that modest to moderate associations between AHI and stroke in women could have been overlooked in the existing data set.

Shahar et al^25^ examined 6424 individuals from the Sleep Heart Health Study (age ≥40 years). When the upper AHI quartile was compared to the lower AHI quartile, the adjusted relative odds ratio for stroke was 1.58 (95% CI, 1.02 to 2.46) after accounting for age, race, sex, smoking status, self-reported diabetes mellitus, total cholesterol, and high-density lipoprotein cholesterol (*P*=0.03). However, when a model was used that added the number of cigarettes smoked per day, self-reported hypertension, systolic blood pressure, use of antihypertensive medications, and body mass index as additional covariates, the model approached but did not reach significance (*P*=0.06; adjusted relative odds ratio 1.55; 95% CI, 0.96 to 2.50). Central apneas were relatively infrequent in the Sleep Heart Health Study, and removal of subjects whose apneic events were central in nature did not alter the findings.

Similar results were reported in other cross-sectional and longitudinal studies.^26–29^ However, central and obstructive apneas were not always separated, and composite endpoints (cardiovascular events and stroke^27–28^ or stroke and death^27–28^) were used. Capampangan and colleagues^30^ used the data available in the study by Yaggi et al^28^ to separate the composite endpoints and calculated a relative risk of 5.16 for the association of OSA with the endpoint (stroke and transient ischemia attack).

Studies involving OSA and small-vessel cerebrovascular disease, including white matter lesions and lacunar infarcts, are limited. Silent brain infarcts, which lack stroke-like symptoms and predominantly involve small-vessel cerebrovascular disease,^31^ were frequent in patients with sleep apnea.^32–34^ Silent brain infarction occurred in 25% of patients with moderate to severe OSA (AHI=45) but occurred in only 6.7% and 7.7% of control subjects (AHI=3) or patients with mild OSA (AHI=11), respectively.^33^ Harbison et al^35^ reported that white matter disease severity correlated with AHI in patients with stroke. Because of the methods of assessing apneas, neither Harbison et al^35^ nor Eguchi et al^32^ were able separate obstructive apnea from central apnea. Others found that small-vessel cerebrovascular disease either was not associated with OSA^36^ or was best correlated with central sleep apnea.^37^

Any relationship between OSA (or sleep-disordered breathing) and small-vessel cerebrovascular disease is complicated by the fact that the risk factors for small-vessel cerebrovascular disease (hypertension, diabetes mellitus, atherosclerosis, and atrial fibrillation) are associated with OSA.^1,5,7,38–42^ Thus, any relationship between OSA and small-vessel cerebrovascular disease could be indirect, through the aforementioned risk factors.

In summary, determining if OSA is an underlying cause of stroke and other cerebrovascular disorders has been a formidable challenge. In studies conducted after the occurrence of stroke, it can be determined only that OSA is more prevalent after stroke but not whether OSA preceded the stroke or was responsible for the stroke. Nevertheless, arguments can be made that preexisting cerebrovascular disease in patients with OSA or snoring (an indicator of OSA) preceded the stroke.^20,23,35^ The best evidence that OSA is an underlying cause of stroke has been derived from cross-sectional and longitudinal studies of the general population. The studies are complicated by confounding comorbidities that might be both the result of OSA on one hand and a risk factor for stroke on the other.^1,5,7,38–39^ However, even after adjustment for these and other confounding comorbidities, OSA was often independently associated with stroke.^24,28–29^ When the literature is considered as a whole, the evidence is compelling that OSA is an independent risk factor for stroke.

### The Effect of OSA on the Outcome After Stroke

OSA not only is a risk factor for stroke but also exacerbates the damage produced by a stroke once it has occurred and increases the risk for a subsequent stroke.^1,7,39,43–46^ OSA in patients with stroke increased the length of hospital stay and the likelihood of death after 6 months, with the most predictive measure for death being the length of apnea.^47^ Sahlin et al^48^ monitored 132 stroke patients over 10 years. Patients with an AHI <15 served as controls; 17% were classified as having OSA; and 21% were classified as having central sleep apnea. Two patients with mixed OSA and central sleep apnea were not included in the analysis. OSA was a significant risk factor for death (adjusted hazard ratio, 1.76; 95% CI, 1.05 to 2.95; *P*=0.03) independent of age, sex, body mass index, current smoking status, hypertension, diabetes mellitus, atrial fibrillation, cognitive impairment, or dependency on caregivers. Interestingly, central sleep apnea was not related to increased rate of death, although AHIs for central and obstructive apnea were similar (33 versus 28, respectively). In addition to contributing to a worse outcome after stroke, sleep apnea (predominantly OSA) was reported to be an independent risk factor for the recurrence of stroke.^43^

Although it is clear that OSA exacerbates the damage produced by a stroke once it has occurred, it is possible that “preconditioning” resulting from repeated episodes of hypoxia prevents the insult from being even worse. Repeated episodes of systemic hypoxia protect the rodent brain from subsequent ischemic damage.^49^ If this observation in rodents can be translated to humans, then preconditioning resulting from repeated hypoxias accompanying OSA could limit the damage of stroke. However, even if preconditioning occurs in the human brain, the existing evidence suggests that the detrimental effects of OSA outweigh any protection from hypoxic preconditioning.

### CPAP Therapy and Stroke

Although some encouraging studies indicate beneficial effects of CPAP treatment in patients with stroke and OSA, the results have not been consistent, and more studies will be required to determine any benefits conclusively.^1,50^ CPAP therapy might be particularly important during the acute stages after stroke in patients with OSA. As a result of stroke, brain regions with compromised rates of blood flow can be affected negatively by ongoing OSA. First, regions with compromised blood flow are further jeopardized by the hypoxia that occurs during OSA. Second, cerebral vessels in healthy tissues more effectively dilate during hypercapnia than do vessels within the compromised regions. During an episode of OSA when hypercapnia occurs, healthy tissue can “steal” blood from regions with inadequate cerebrovascular reserve. This phenomenon is called the “Reversed Robin Hood Syndrome.”^51–53^ Thus, CPAP therapy in OSA patients could be especially important during the acute phase after stroke by reducing or preventing apneas and possibly preventing further damage to brain regions at risk because of compromised blood flow.^54–55^

**Figure 2.**
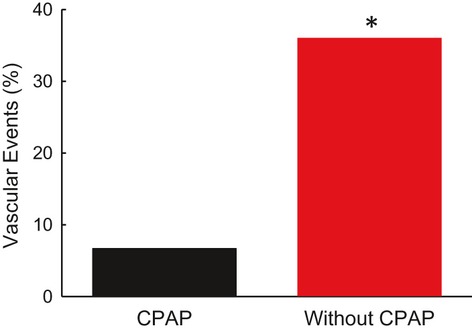
Incidence of vascular events over an 18-month period after stroke in CPAP users (n=15) and those who discontinued the use of CPAP (n=36). All patients in the study had an AHI ≥20. *P=0.03. Created with data from Martinez-Garcia et al.^56^

Martinez-Garcia et al^56^ studied the effects of CPAP therapy in OSA patients after ischemic stroke. Two months after the acute stroke, patients were assessed for the presence of sleep apnea. Only patients with AHI ≥20 were enrolled, and CPAP therapy was prescribed for all patients. The patients were divided into 1 of 2 groups: those who tolerated CPAP therapy for the duration of the study (15 patients) and those who could not tolerate and discontinued CPAP therapy after 1 month (36 patients). The incidence of new cerebrovascular or ischemic coronary events was determined during the subsequent 18 months. The incidence of new vascular events was 5-fold greater in patients who discontinued CPAP than in CPAP users after adjustment for other vascular risk factors and neurological indexes (Figure 2). Martinez-Garcia et al concluded that CPAP treatment in patients with mild to severe sleep apnea can offer protection against new vascular events after ischemic stroke. Wessendorf et al^57^ reported an improved sense of well-being and decreased blood pressure after 10 days in stroke patients (n=74) who adhered to CPAP therapy compared to those who did not (n=36). Ryan et al^57a^ randomized stroke patients with OSA (AHI ≥15) to a CPAP (n=22) group or control (n=22) group. CPAP treatment for 4 weeks improved functional and motor outcomes but not neurocognitive outcomes. The authors concluded that CPAP treatment provided a “significant, although modest, beneficial effect” on stroke-related outcomes in patients with OSA. However, another study found no beneficial effects of 8 weeks or 6 months of CPAP therapy in stroke patients with AHI ≥30.^58^ However, in that study, patients in the treatment group had limited compliance with CPAP, with the average use being only 1.4 hours per night. Depending on the study, compliance with CPAP therapy among patients who had a stroke was similar to or lower than compliance in patients who did not have a stroke.^57–61^

### OSA, Dementia, and Cognitive Impairment

Published studies have reported an association between OSA and dementia in the elderly,^62–63^ with the severity of the dementia being related to the severity of the sleep-disordered breathing.^64–65^ In one study, Yaffe et al^66^ monitored 298 women without dementia (mean of 82 years of age) for ≍5 years. Women with sleep-disordered breathing were more likely to develop mild cognitive impairment or dementia even after adjustment for other risk factors. The vast majority of the dementia and cognitive impairment in individuals >80 years of age is mixed, as opposed to pure Alzheimer's disease or vascular in nature.^67^ Given that OSA might be an underlying cause of stroke and that strokes can be a major component in brain atrophy and confusion in older patients,^68^ it stands to reason that OSA, though not necessarily the underlying cause of the dementia, is certainly a factor that would exacerbate the dementia.^67^

Ayalon et al^69^ assessed cognitive function in younger (25 to 44 years) and older (45 to 59 years) subjects with and without OSA. Older subjects with OSA showed cognitive decline when compared to older subjects without OSA, younger subjects with OSA, or younger subjects without OSA. Thus, cognitive decline, which normally occurs with aging, can be accelerated in individuals with OSA.

Studies indicate that patients with OSA and Alzheimer's disease could have positive benefits from CPAP therapy. Modest but statistically significant cognitive improvements were reported after 3 weeks of CPAP therapy in one study.^70^ CPAP treatment for 13 months showed positive benefits in mood, sleep quality, and cognition compared to a group of patients not using CPAP treatment.^71^ Although definitive conclusions are hampered by the limited sample sizes, the findings are still provocative and should motivate future studies. As such, CPAP therapy might provide lasting improvements, or at least stabilization, in the quality of life of individuals with dementia.

If the studies are taken as a whole, a convincing argument can be made that OSA accelerates cognitive decline and the onset and severity of dementia,^62–66^ although not all studies found a significant association between OSA and cognitive function in the elderly.^72–73^ That the incidence of OSA increase with age^14^ adds to the seriousness of the clinical situation in the elderly.

## Effects of OSA on the Cerebral Circulation

The cerebrovascular system must accommodate the distinct anatomic structure and physiological function of the brain. As a consequence, it is different in many respects from the peripheral vascular system. These differences present unique challenges and vulnerabilities for the cerebrovascular system in maintaining a homeostatic environment in which the brain can operate. The O_2_ demand by the brain requires an uninterrupted supply of O_2_ by the blood. Disruptions in O_2_ delivery for relatively short periods of time are accompanied by significant pathological consequences. In this review, we will discuss only a few aspects of the cerebral circulation as they pertain to OSA. For in-depth discussion of the cerebral circulation, see the referenced reviews.^74–78^

## Cerebral Blood Flow During an Episode of Apnea

Transcranial Doppler ultrasonography (TCD) measurements of blood velocity in cerebral arteries have been an important tool in assessing cerebral blood flow (CBF) in humans. TCD, which measures the velocity of red blood cells in vessels (usually the middle cerebral artery), does not provide a quantitative measure of CBF. Instead, it provides an index that is directly proportional to the rate of blood flow, provided the diameter of the vessel remains constant throughout the time of measurement. Several studies indicate that CBF velocity (CBFV) in the middle cerebral artery can provide a reasonable index of CBF with different conditions.^79–81^ Although TCD has its limitations, it is a powerful and noninvasive tool that has been used in humans to provide meaningful insight into changes in CBF during OSA.

**Figure 3.**
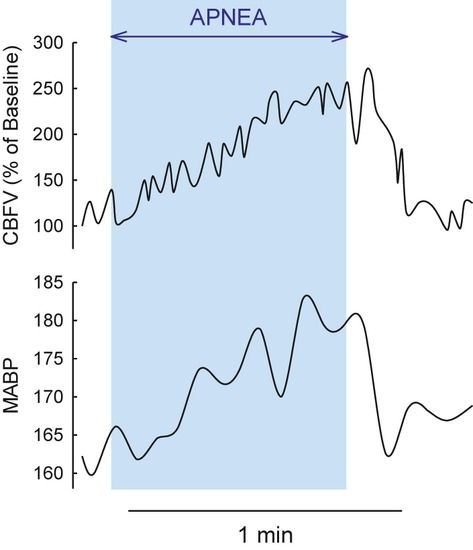
Changes in blood flow velocity in the middle cerebral artery (CBFV; top) and mean arterial blood pressure (MABP; bottom) during an episode of apnea (shaded in light blue). Created with data from Klingelhofer et al.^83^

CBFV has been reported to increase steadily in the middle cerebral artery during an episode of OSA. The magnitude of the CBFV increase varies greatly but can exceed 200% of the baseline velocity (Figure 3).^5,82–84^ On termination of apnea, CBFV often, but not always, decreases below baseline before recovering after ≍1 minute.^5,82,84^

Several known, and likely some unknown, vasoactive influences impinge on the cerebral circulation during OSA. During an episode of OSA, arterial Po_2_ and pH decrease, and Pco_2_ increases.^1,7^ Blood gasses and pH changes in these directions act in concert to dilate cerebral arteries and arterioles.^85–86^ The increase in arterial Pco_2_ (hypercapnia) during an episode of OSA has been more of a focus on CBF regulation than the concomitant hypoxia.^83,87–88^

Arterial blood pressure has been reported to increase progressively during the apnea and to fall below baseline after the apnea.^5,82–83^ If autoregulation is compromised (see later) or the changes in blood pressure are sufficiently rapid that autoregulation, even if intact, does not have time to adapt, then all or a portion of the changes in TCD velocity could be related to the changes in blood pressure.^5^ Indeed, Balfors and Franklin^82^ reported a linear correlation between percent change in mean arterial blood pressure and percent change in CBFV during and after an episode of apnea. It must be noted that during an episode of OSA, blood pressure mostly increases, Po_2_ and pH decrease, and Pco_2_ increases. Therefore, it is not surprising that changes in the CBFV also correlate with arterial Pco_2_.^83,88^

Several publications in the literature present CBFV and O_2_ saturation data during a single or multiple OSA event(s).^82,84,89^ The timing of maximal O_2_ desaturations often occurred after breathing had resumed. For example, Balfors and Franklin^82^ noted that maximum O_2_ desaturation (as assessed by pulse oximetry) occurred ≍20 seconds after termination of the apnea. Although the delay was noted in the previously discussed studies, no explanations were provided for the unexpected timing of the O_2_ desaturations. We suggest that the timing of the O_2_ desaturations shown in these publications represents a delay in the output of the measuring devices. The delay time for pulse oximetry, which depends on manufacturer, sampling interval, and probe placement, can be ≥30 seconds.^90–91^

Contrary to the majority of studies that show an increase in CBFV during OSA, Netzer et al^92^ reported that CBFV decreased during 76% of obstructive hypopneas and 80% of obstructive apneas observed. The reason for this disparity in the literature is not known. However, blood pressure was not measured in the study by Netzer et al.^92^ As discussed previously, changes in arterial blood pressure can be an important consideration in CBF changes during an episode of OSA.

Other changes that potentially could affect CBF during an episode of OSA include vasoactive substances in blood or stimulation of vasoactive central neurons.^93–94^ In addition, the sensory, sympathetic, or parasympathetic nervous system innervates cerebral arteries^95^ and could influence CBF during an episode of OSA. In general, however, activation of the sympathetic nervous system has little effect on the cerebral circulation except during some pathological states.^95–96^ Although none of the systems that innervate cerebral vessels has been investigated in OSA, they must be considered, given the complexity and the number of systems involved with the response to OSA.

In healthy awake subjects, the CBFV increases observed during voluntary apnea and the CBFV decreases observed after the apnea were driven largely by arterial Pco_2_ and to a lesser extent by changes in arterial pressure.^87^ Although these studies in healthy subjects could provide insight into CBF during OSA, it must be pointed out that the mechanisms in healthy subjects might not totally reflect the mechanisms in individuals with OSA. The increase in CBFV during voluntary apnea was reduced in patients with OSA compared to control subjects. Significantly, the response to the voluntary apnea returned to normal after 1 day or 1 month of CPAP therapy.^97–98^

Impaired cerebrovascular control in individuals with OSA can affect the energy state of the brain during an episode of apnea. When O_2_ desaturations were >10% during OSA, brain adenosine triphosphate decreased, and inorganic phosphate increased.^99^ With different protocols, comparable or even greater levels of hypoxia did not significantly alter the energy state in normal subjects or in animal models.^100–102^ During nonpathological conditions, an increase in CBF can accommodate the O_2_ requirement for adenosine triphosphate production through the respiratory chain. Rae et al^99^ concluded that “transient hypoxia experienced during sleep may impair brain function more than previously thought.”

## Effects of OSA on Resting CBF

Although they are limited, studies indicate that resting CBF is chronically altered in individuals with OSA. Meyer and colleagues^103–104^ reported that CBF in patients with OSA was decreased during both sleep and wakefulness compared to volunteers without OSA. These studies, using stable xenon and xenon-133 computerized tomography, are significant in that they are the only studies, to our knowledge, in which rates for CBF (mL / 100 g per minute) have been actually quantified in patients with OSA. CBF was decreased in the hemispheric, brainstem, and cerebellar gray regions of patients with OSA during wakefulness.^103–104^ Additionally, during sleep, patients with OSA exhibited significant reductions in flow to the frontal and occipital cortex, pons, and the cerebellum.^103^ These decreases occurred even though end-tidal CO_2_ was significantly greater in patients with OSA during wakefulness (2 mm Hg) and sleep (4 mm Hg) than in control subjects.^103^

TCD studies support the idea that resting CBF is decreased in individuals with OSA.^105^ This decrease in CBFV persisted in wakefulness, non–rapid eye movement sleep, and rapid eye movement sleep in patients with OSA, despite their having end-tidal CO_2_ values greater than those of control subjects for all states.^106^ Hajak et al^107^ extended these findings by measuring CBFV during the 4 different stages of non–rapid eye movement sleep. CBFV was decreased in patients with OSA during sleep stages 1 to 3 after falling asleep compared to control subjects. However, during all stages of rapid eye movement sleep and in most sleep stage twos after the first occurrence, CBFV was greater in patients with OSA than in controls. Overall, patients with OSA and control subjects exhibited similar patterns of fluctuations in nocturnal brain perfusion; however, the amplitude of these changes was greater in control subjects during non–rapid eye movement sleep and was greater for patients with OSA during rapid eye movement transitions.^107^

When the data are taken as a whole, it can be concluded that CBF is decreased in the resting state with OSA. Although there are some variations between studies, particularly during sleep, existing studies consistently report alterations in resting CBF. The resting state reflects the background upon which CBF is regulated, and deviations from the resting state of healthy individuals are indicative of cerebrovascular dysfunction. Of note, moderate decreases in CBF can have significant adverse consequences, including deficits in memory, spatial learning, and attention, which are described commonly in patients with severe OSA.^108–111^ Consistent with this idea is a recent study that shows disruption of neurovascular coupling in the sensory cortex in mice after intermittent hypoxia, a model of sleep apnea.^112^

## Effects of OSA on Cerebral Autoregulation

Autoregulation helps to maintain a homeostatic environment in which the brain can operate by maintaining a constant CBF in the face of changes in arterial blood pressures over a range between 60 and 160 mm Hg.^96^ Unlike many peripheral tissues in which the major resistance to flow resides in the arterioles, the resistance in the cerebrovascular system occurs along the entire vascular tree, including not only the smaller arteries and arterioles but also the larger cerebral arteries.^109,113–114^ The vascular tree in a healthy brain, in which large arteries contribute significantly to the resistance, not only serves to regulate CBF but also protects arterioles and capillaries from excessive pressures.

**Figure 4.**
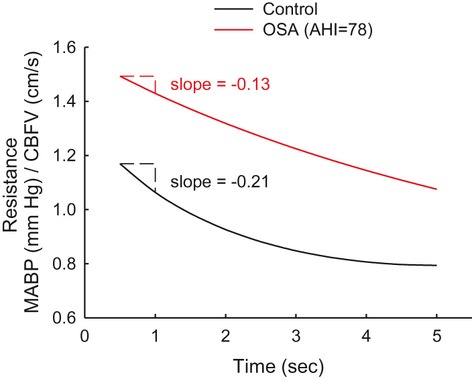
Resistances to blood flow through the middle cerebral artery in control subjects (n=26) and patients with OSA (n=78) after an orthostatic hypotension challenge, obtained by having individuals stand from a squatting position.^105^ The patients with OSA had a mean AHI of 78. The resistance was calculated by dividing mean arterial blood pressure (MABP) by blood flow velocity in the middle cerebral artery (CBFV). The increased resistance in the patients with OSA during orthostatic hypotension indicates that autoregulatory mechanisms responsible for dilating cerebral arteries were impaired relative to the control subjects. The response of the cerebral circulation to the hypotensive challenge was less dynamic in patients with OSA during the initial phase of the hypotensive challenge. That is, the rate of change in resistance in patients with OSA was only 62% of that in control subjects (100×[−0.13/−0.21]). Created with data from Urbano et al.^105^

Urbano et al^105^ exposed control subjects and patients with OSA (average AHI=78) to an orthostatic hypotension challenge by having each subject stand from a squatting position. The recovery of CBFV in the middle cerebral artery after a transient decrease in mean arterial blood pressure was used to assess the autoregulatory response. The resistance to flow in the middle cerebral artery (calculated as mean arterial blood pressure / CBFV) was greater in patients with OSA after standing than in control subjects (Figure 4). The increased resistance in the patients with OSA during orthostatic hypotension indicates that autoregulatory mechanisms responsible for dilating cerebral arteries were impaired relative to those in control subjects. In addition, the response of the cerebral circulation to the hypotensive challenge was less dynamic in patients with OSA during the initial phase of the hypotensive challenge, being only 62% of that in control subjects (slopes in Figure 4). Nasr et al^115^ studied the autoregulation in awake patients with moderate OSA (average AHI=23) and matched controls by computing an autoregulatory index consisting of a moving correlation coefficient between mean CBFV and mean arterial blood pressure. Not only was autoregulation impaired in awake patients with OSA, but also the severity of the impairment strongly correlated with OSA severity.^115^ Finally, a recent study showed that the mechanism for myogenic tone, the intrinsic property of arteries to constrict when pressurized, was altered in ex vivo middle cerebral arteries from rats after they had been subjected to intermittent hypoxia.^116^ An impairment of cerebral autoregulation likely contributes to the increased incidence of stroke, as well as the poor outcome after stroke, in patients with OSA.

## Effects of OSA on Cerebrovascular Response to Hypoxia and Hypercapnia

Repeated exposures to hypoxia in individuals with OSA could lead to hypoxic/ischemic brain injury, especially if cerebrovascular control is impaired. The increase in CBFV in the middle cerebral artery during hypoxic challenges was attenuated significantly by 42%^117^ and 36%^118^ in patients with severe OSA as compared to controls. The CBFV response to hypoxia correlated with the AHI and nocturnal O_2_ desaturation.^117^ Interestingly, significant improvements in the CBFV response to hypoxia occurred after 4 to 6 weeks^117^ or 12 weeks^118^ of CPAP treatment. Finally, the dilator response to hypoxia in isolated pressurized middle cerebral arteries from rats after 2 weeks of intermittent hypoxia was completely abolished.^119^

Studies investigating the cerebrovascular response to hypercapnia in patients with OSA have yielded conflicting results. Studies performed by Urbano et al and Foster et al^105,120^ exposed patients with severe OSA to repetitive hypercapnic conditions while measuring CBFV by TCD. Both studies reported no difference in the hypercapnic response as compared to control patients. Reichmuth et al^118^ reported that the increase in CBFV when end-tidal CO_2_ was increased by 5 and 10 mm Hg was attenuated by 22% in individuals with OSA compared to controls. Similarly, Morgan et al^121^ reported that the cerebrovascular response to hypercapnia was blunted in individuals with OSA, with the severity of the response positively correlating with the degree of oxygen desaturation during OSA. Using methods to quantitatively measure CBF, Meyer et al^103–104^ reported that the increase in CBF with the addition of 5% CO_2_ to the ventilator gas was blunted in patients with OSA during sleep and wakefulness compared to control subjects. Given the limited number of studies and the conflicting results, any conclusion about the cerebrovascular response to hypercapnia is premature. Unlike the autoregulatory response and the cerebrovascular responses to hypoxia, the effects of OSA on the response to hypercapnia must await further investigations.

## Mechanisms of Injury to the Cerebral Circulation in OSA

### Physical Mechanisms

Several events take place during an episode of apnea, in addition to hypoxia/reoxygenation, that have detrimental effects on the cerebral circulation. There are repeated swings in blood pressure with each episode of apnea, consisting not only of increased blood pressure during the episode but also a period of hypotension after the episode.^82–83^ At the end of an episode of OSA, blood pressure was reported to sharply increase by ≍35 mm Hg on average; however, in some individuals, the systolic pressure surge exceeds 100 mm Hg.^1,122^ These surges in blood pressure can be sufficiently rapid that autoregulation, even if it were intact, might not have time to fully accommodate.^5^ Pressure surges will expose smaller arteries, arterioles, and capillaries to damaging pressures, resulting in endothelial damage and disruption of the blood–brain barrier. At the end of an episode in which the blood pressure sharply increased, intracranial pressure was reported to often exceed 50 mm Hg.^123^ In a similar manner, the sharp decreases in blood pressure on termination of OSA^82–83^ can leave the brain vulnerable to ischemia, especially in regions with poor cerebrovascular reserve.^39^ This would be especially true for areas without good collateral circulation, including border zone areas and terminal arterial territories. Consistent with this idea is the fact that OSA has been associated with lacunar infarcts, small-vessel disease, and leukoaraiosis.^33,35,124^

Although the carotid artery is outside the cranial vault, the carotid artery must be included when CBF is considered because the majority of blood flow to the brain is via the carotid arteries. The carotid arteries are a primary site for development of atherosclerosis (carotid artery disease), a risk factor for stroke.^109^ Vibrations in the carotid arteries from snoring, common in patients with OSA, can produce endothelial damage and atherosclerosis.^125–126^ Vascular dysfunction in the carotid artery will put more stress on the vascular system in the brain, rendering it more vulnerable to other pathological consequences of OSA.

### Cellular and Molecular Mechanisms

There is a paucity of studies involving pathological mechanisms in the cerebral circulation in OSA. Given the more extensive literature involving vascular effects outside of the brain (see reviews^1,3,7,9,16,127–131^), it is tempting to extrapolate these findings directly to the cerebrovascular circulation. However, extrapolation must be conducted with caution because (1) the distinct anatomic structures and physiological function of the brain are uniquely affected by disease processes and (2) the cerebrovascular circulation can be more sensitive to the pathological processes.^109^

OSA initiates a pathological cascade through multiple and often redundant pathways affecting the cardiovascular system (Figure 1).^1^ This cascade includes oxidative stress, inflammation, sympathetic activation, hypercoagulation, endothelial dysfunction, increased platelet aggregation, and metabolic dysregulation.^9,26,38–39,108^ As with the peripheral circulation, the cerebral circulation is affected adversely by the pathological cascade, which impinges to a great extent on the endothelium. The pathological impact on the endothelium, commonly referred to as endothelial “dysfunction,” is characterized by oxidative stress and decreased production of nitric oxide (NO), although the full extent of endothelial dysfunction is much more involved.^9^ Notably, dysfunction of cerebrovascular endothelium occurs sooner and to a greater extent in the progression of cardiovascular diseases than that of endothelium in the periphery.^109^

The sympathetic nervous system is activated acutely during OSA as a result of (1) chemoreceptor discharges with changes in blood gasses and pH, (2) arousals from sleep, and (3) sleep deprivation.^1,7^ In addition to the acute activation during a single episode, chronic intermittent hypoxia or OSA alters the sensitivity of the chemoreceptors and baroreceptors, as well as altering their central integration by nuclei in the brainstem and hypothalamus.^3,132–136^ The carotid chemoreceptors increase baseline activity and become more sensitive to hypoxia through alterations in neurotransmitter systems, including endothelin-1 (ET-1) and angiotensin II.^135,137–139^ The mechanism for altered chemoreceptor function involves reactive oxygen species (ROS), resulting from transcriptional genes that upregulate hypoxia-inducible factor-1 and downregulate hypoxia-inducible factor-2.^135^

**Figure 5.**
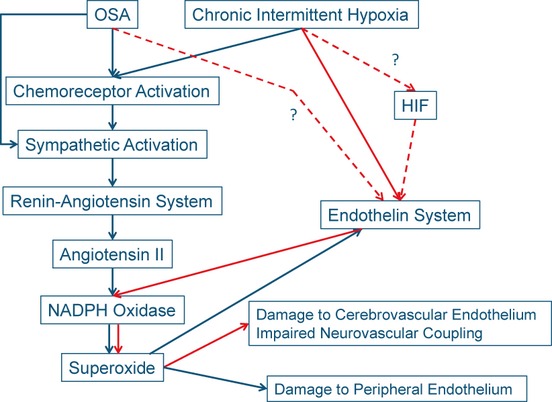
Proposed pathway for activation of NADPH oxidase, generation of superoxide, and upregulation of the endothelin system in the peripheral circulation after OSA and chronic intermittent hypoxia (black lines). An alternative pathway that could be responsible for generation of superoxide in the cerebral circulation (red lines).^112^ Dashed red lines (and “?”), which lack any experimental evidence, represent hypothetical pathways. HIF indicates hypoxia inducible factor.

One consequence of increased sympathetic activity on the kidney is stimulation of the renin–angiotensin system and the subsequent increase in angiotensin II. Angiotensin II can enhance oxidative stress, produce inflammation, disrupt blood–brain barrier integrity, initiate vessel remodeling, and impair increases in nutritive blood flow.^109,140–142^ Through stimulation of angiotensin I receptors on the endothelium, angiotensin II enhances the production of superoxide by activating NADPH oxidase.^143^ Although angiotensin II has similar effects in all endothelial cells, cerebral vessels have greater NADPH oxidase activity than do peripheral vessels and are particularly sensitive to the effects of the superoxide generated.^109,144–146^ The pathway described in this paragraph is shown in Figure 5 (black lines). Although it is not known whether this particular pathway affects the cerebral circulation after chronic OSA, other pathological states negatively impact the cerebral circulation through activation of the renin–angiotensin system, upregulation of NADPH oxidase, and generation of superoxide.^109^ In addition to NADPH oxidase being a major source, superoxide also can be generated in the vascular system by mitochondria, cyclooxygenase, lipoxygenases, hydroxylases, and xanthine oxidase.

Superoxide is involved in the generation of other ROS and reactive nitrogen species. One of these reactive nitrogen species, peroxynitrite, is produced when superoxide reacts with NO. Peroxynitrite uncouples endothelial NO synthase, whereby O_2_ becomes the terminal electron acceptor in the oxidation-reduction reaction to create superoxide in place of the normal end product, NO.^1,7,109^ The net result of an increased oxidative state includes reduced NO bioavailability, damage to lipid membranes, and alterations in protein structure and function. In addition to the increased production of ROS, reduced glutathione, a component of the antioxidant system for protecting the brain, was reported to be significantly reduced with reoccurring obstructive apneas over 1 hour, whereas reduced glutathione in the periphery was not affected.^147^

Although studies of endothelial dysfunction in cerebral arteries are limited, intermittent hypoxia, a model for sleep apnea, produced endothelial dysfunction in rodents.^112,119^ After only 2 weeks of intermittent hypoxia in rats, dilations to acetylcholine in isolated middle cerebral arteries were attenuated by ≍90%.^119^ Activation of cholinergic receptors on the endothelium stimulate the synthesis of NO by endothelial NO synthase. Once generated in the endothelium, NO diffuses to the vascular smooth muscle and activates guanylyl cyclase, the initial step in a dilatory pathway. Phillips et al^119^ demonstrated that intermittent hypoxia did not alter the dilations when guanylyl cyclase was activated directly by sodium nitroprusside. Thus, attenuated dilations after intermittent hypoxia were the result of reduced NO (ie, endothelial dysfunction) because the vascular smooth muscle still had the ability to dilate.

In a recent study, Capone et al^112^ amplified and extended the studies by Phillips et al.^119^ Increases in cortical blood flow in response to dilators requiring intact endothelium were attenuated by ≍40% after 14 and 35 days of intermittent hypoxia in mice. However, dilations in response to adenosine, which acts directly on the vascular smooth muscle, were not affected by intermittent hypoxia. ROS was enhanced significantly in both cerebral arterioles and neurons after intermittent hypoxia, as compared to tissues from control mice.^112^ Significantly, an acute application of a superoxide dismutase mimetic to decrease the oxidative stress completely restored the blood flow response to acetylcholine.^112^ The source of the ROS after the intermittent hypoxia was predominantly, if not exclusively, from superoxide generated by NADPH oxidase, because an inhibitor of NADPH oxidase or mice lacking functional NADPH oxidase-2 showed neither endothelial dysfunction nor enhanced tissue ROS after 35 days of intermittent hypoxia. The studies by Capone et al^112^ complement previous studies demonstrating a crucial role for brain NADPH oxidase in hypersomnolence and the decline in cognitive function after intermittent hypoxia or sleep fragmentation.^148–150^ Thus, intermittent hypoxia produces oxidative stress and decreases the bioavailability of endothelium-derived NO through superoxide derived from NADPH oxidase-2.

Crossland et al^151^ developed a model of OSA in rats in which apnea was elicited in unanesthetized freely ranging rats by remotely inflating a balloon implanted in the trachea. The balloon was inflated for 10 seconds 30 times per hour during 8 hours of the sleep cycle every day for 28 days. Dilations in isolated pressurized middle cerebral arteries, elicited by stimulating endothelial P2Y_2_ receptors by adenosine triphosphate, were attenuated by ≍40% compared to a control group that was surgically prepared but did not undergo apnea. Although the dilation in response to adenosine triphosphate was the result of multiple endothelial-dependent mechanisms of dilation,^152–155^ the attenuated response was due to suppression of the NO component.^151^ Thus, there is continuity with the findings from different animal models for OSA, intermittent hypoxia, and apnea.

ET-1 is a potent vasoconstrictor that is involved with pathological processes, including OSA. The primary, but not exclusive, source for ET-1 in the vascular system is endothelial cells^156–157^; however, during pathological states, the vascular smooth muscle also can synthesize ET-1.^156–157^ Stimulation of endothelin receptor type B on the endothelium produces dilation of vessels through generation of NO, whereas stimulation of primarily endothelin receptor type A, but sometimes endothelin receptor type B, on the vascular smooth muscle produces potent and sustained constrictions.^156,158^ During normal physiological states, NO inhibits transcription and release of ET-1, reduces the interaction of ET-1 with endothelin receptors on smooth muscle, and interferes with second messenger signaling in the endothelin signaling pathway.^159–160^ However, during pathological states such as OSA, reduced NO bioavailability, as a result of the oxidative stress, allows the endothelin pathway to upregulate. The endothelin system further adds to the oxidative stress by enhancing the pathological cascade that generates ROS.^161–164^

The endothelin system is upregulated in animal models of OSA and likely is upregulated with OSA in humans, although plasma ET-1 levels might not be increased.^1,3,9,16,38,112,127,165–168^ Intermittent hypoxia increased ET-1 in cerebral vessels of mice by ≍2 orders of magnitude and increased mRNA for endothelin-converting enzyme-1 and endothelin receptor type A but not mRNA for endothelin receptor type B.^112^ Furthermore, the increase in ET-1 expression occurred in both the endothelium and vascular smooth muscle. The attenuated cerebrovascular responses to acetylcholine with intermittent hypoxia were restored after acutely blocking endothelin type A receptors.^112^ Administration of the endothelin type A blocker also reduced ROS in cerebral vessels and neurons after intermittent hypoxia to the levels in control mice.^112^ It is interesting to note that either inhibiting NADPH oxidase-2 or blocking endothelin type A receptors reduced ROS and restored the cerebrovascular responses to endothelial-mediated dilations. Upregulation of NADPH oxidase often is associated with angiotensin II; however, with intermittent hypoxia, it seems that ET-1 replaces the role of angiotensin II in regulating NADPH oxidase. This alternative pathway is shown in Figure 5 (red lines). It is possible that the endothelin system is upregulated during chronic intermittent hypoxia by a hypoxia-inducible factor.^169^ However, at this time, we do not know if inhibition of NADPH oxidase or scavenging ROS prevents upregulation of the endothelin system in the context of intermittent hypoxia. Answers to these and other significant questions are needed for a clear understanding of the relationships among NADPH oxidase, ROS, and the endothelin system.

**Figure 6.**
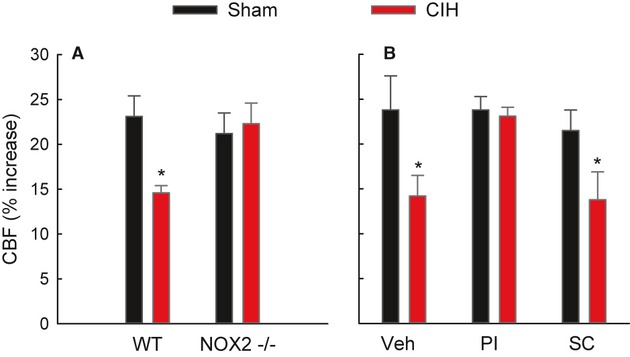
A, The effects of 35 days of chronic intermittent hypoxia (CIH) on increases in laser Doppler perfusion (CBF) in the sensory cortex accompanying whisker stimulation. Sham mice were not exposed to CIH. A, In wild-type mice (WT) and mice lacking NADPH oxidase-2 (NOX2−/−). B, After administration of vehicle (Veh), a peptide inhibitor of NADPH oxidase (PI), or a scrambled control peptide (SC). *P<0.05. Created with data from Capone et al.^112^

Capone et al^112^ also demonstrated that the increases in cerebral perfusion resulting from sensory input (ie, neurovascular coupling) were attenuated after intermittent hypoxia. As with dilations involving stimulation of endothelial receptors, neurovascular coupling could be restored by inhibition of NADPH oxidase signaling (Figure 6), reduction of the oxidative state, or blocking of endothelin type A receptors.^112^ Attenuation of neurovascular coupling implicates dysfunction in cell types other than the endothelium because it involves communications among neurons, astrocytes, and vascular smooth muscle.^86^ Thus, OSA, or at least intermittent hypoxia, affects not only the endothelium but also the entire neurovascular unit.

## Summary

OSA is a serious condition that is the cause of cardiovascular disease or increases the severity and progression of cardiovascular disease. Strong evidence indicates that (1) OSA is an independent risk factor for stroke, (2) OSA exacerbates damage produced by a stroke, (3) OSA increases the risk for a subsequent stroke, and (4) OSA contributes to brain atrophy and dementia in the elderly. OSA interferes with the basic control mechanisms for regulating CBF by decreasing resting CBF, impairing autoregulation, and reducing cerebrovascular reserve. These alterations to normal cerebrovascular control interfere with brain function and render the brain more vulnerable to ischemic events. Although we only have begun to understand the molecular and cellular mechanisms involved with cerebrovascular dysfunction in OSA, endothelial dysfunction, oxidative stress, and the endothelin system seem to have major roles. Given that OSA can continue for years without recognition and that most individuals with OSA go undiagnosed, it is logical that the damage to the cerebral circulation occurs over a long period of time, creating an environment that predisposes an individual to stroke, transient ischemia attacks, and dementia. Because our population is aging and becoming more obese (risk factors for OSA), the incidence of cerebrovascular disease associated with OSA should be expected to increase, and cerebrovascular disease should occur at an earlier age.
